# Mutacin 1140 Lantibiotic Variants Are Efficacious Against *Clostridium difficile* Infection

**DOI:** 10.3389/fmicb.2018.00415

**Published:** 2018-03-16

**Authors:** Johan A. Kers, Robert E. Sharp, Anthony W. Defusco, Jae H. Park, Jin Xu, Mark E. Pulse, William J. Weiss, Martin Handfield

**Affiliations:** ^1^Industrial Products Division, Intrexon Corp., South San Francisco, CA, United States; ^2^Oragenics, Inc., Tampa, FL, United States; ^3^Department of Chemistry, University of Massachusetts Lowell, Lowell, MA, United States; ^4^PreClinical Services, UNT System College of Pharmacy, Fort Worth, TX, United States

**Keywords:** lanthipeptide, antibiotic, bacteriocin, nisin, mutagenesis, structural variant, resistance

## Abstract

Lantibiotics offer an untapped pipeline for the development of novel antibiotics to treat serious Gram-positive (+) infections including *Clostridium difficile*. Mutacin 1140 (MU1140) is a lantibiotic produced by *Streptococcus mutans* and acts via a novel mechanism of action, which may limit the development of resistance. This study sought to identify a lead compound for the treatment of *C. difficile* associated diarrhea (CDAD). Compounds were selected from a saturation mutagenesis library of 418 single amino acid variants of MU1140. Compounds were produced by small scale fermentation, purified, characterized and then subjected to a panel of assays aimed at identifying the best performers. The screening assays included: *in vitro* susceptibility testing [MIC against *Micrococcus luteus*, *Clostridium difficile*, vancomycin-resistant enterococci (VRE), *Staphylococcus aureus*, *Streptococcus pneumonia*, *Mycobacterium phlei*, and *Pseudomonas aeruginosa*; cytotoxicity screening on HepG2 hepatocytes; *in vitro* pharmacological profiling with the Safety Screen 44^TM^, metabolic and chemical stability in biologically relevant fluids (FaSSGF, FaSSIF and serum); and efficacy *in vivo*]. Several lantibiotic compounds had better MIC against *C. difficile*, compared to vancomycin, but not against other bacterial species tested. The Safety Screen 44^TM^ *in vitro* pharmacological profiling assay suggested that this class of compounds has relatively low overall toxicity and that compound OG253 (MU1140, Phe1Ile) is not likely to present inadvertent off-target effects, as evidenced by a low promiscuity score. The *in vitro* cytotoxicity assay also indicated that this class of compounds was characterized by low toxicity; the EC_50_ of OG253 was 636 mg/mL on HepG2 cells. The half-life in simulated gastric fluid was >240 min. for all compound tested. The stability in simulated intestinal fluid ranged between a half-life of 5 min to >240 min, and paralleled the half-life in serum. OG253 ultimately emerged as the lead compound based on superior *in vivo* efficacy along with an apparent lack of relapse in a hamster model of infection. The lessons learned from this report are applicable to therapeutic lanthipeptides in general and may assist in the design of novel molecules with improved pharmacological, therapeutic and physicochemical profiles. The data presented also support the continued clinical development of OG253 as a novel antibiotic against CDAD that could prevent recurrence of the infection.

## Introduction

The Centers for Disease Control (CDC) report on Antibiotic Resistance Threats in the United States (Centers for Disease Control and Prevention [CDC], 2013) classifies *Clostridium difficile* infection as an urgent threat, based on the high incidence of 250,000 infections per year and a mortality rate of 14,000 deaths per year. Due to increasing rates of mortality and the emergence of strains with increased virulence, it is anticipated that the current cost of medical care, which currently exceeds $1 billion per year in the US alone, will continue to increase. Although the epidemiology of *C. difficile* infection (CDI) can vary markedly in different countries, with many experiencing high rates of CDI and general incidence rates that are increasing (Wilcox, 2016). This crisis has ushered in national programs aimed at increasing antibiotic stewardship and recently added new drugs/biologicals in the pharmacopeia. In 2011, the FDA/EMA approved fidaxomicin (Dificid) (Vaishnavi, 2015), while the antitoxin antibody bezlotoxumab (Zinplava) was recently approved by the FDA and the EMA in 2016 and 2017, respectively (Kufel et al., 2017). While new options for infectious disease treatment have been welcomed, there is concern that their high cost may prevent wide-spread use (Surawicz et al., 2013). Nevertheless, there continues to be extensive research efforts due to the high unmet clinical need to develop additional novel strategies to combat *C. difficile* infections, as presented in this special Research Topic Issue, including novel small molecule and peptide-derived antimicrobials, and extending to alternative approaches such as fecal transplantation (FMT), microbial restoration and phage therapy, *etc*.

**FIGURE 1:**
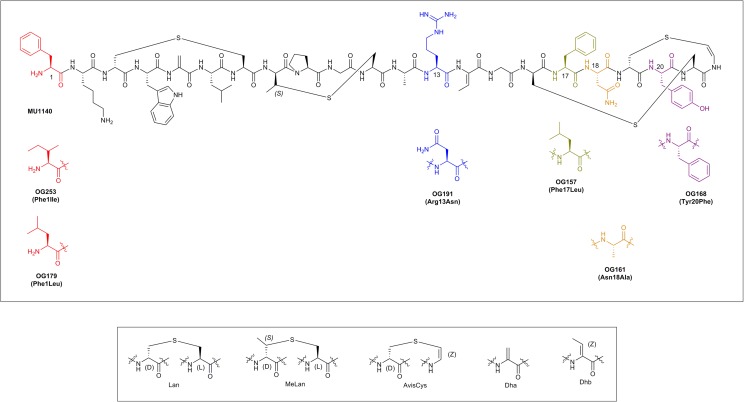
The chemical structure of MU1140 and top 6 variants (top). Substitutions are displayed in parenthesis. Post-translationally modified residues in lantibiotics are presented in the (bottom).

Lantibiotics are a relatively old class of compounds that were initially discovered around the same time as penicillin; the archetype lantibiotic nisin was first discovered in 1928 and subsequently isolated in 1947 (Rogers and Whittier, 1928; Field et al., 2015). Lantibiotics are part of a larger class of lanthipeptides that derive their name from the thioether ring containing amino acids lanthionine (Lan, Ala-S-Ala) and/or 3-methyl-lanthionine (MeLan, Abu-S-Ala). These compounds often incorporate post-translationally modified amino acids such as Dha, Dhb, and the unsaturated lanthionine derivatives AviCys at their *C*-terminus (reviewed in Chatterjee et al., 2005; Smith and Hillman, 2008; Ross and Vederas, 2011; see **Figure 1** and **Table 1**).

**Table 1:** Amino-acid sequence of the core peptide precursor and post-translationally modified MU1140.

Precursor	Ser3	Ser5	Cys7	Thr8	Cys11	Thr14	Ser16	Ser19	Cys21	Cys22

Modified	**Ala3**	Dha5	**Ala7**	**Abu8**	**Ala11**	Dhb14	**Ala16**	**Ala19**	**Ala21**	**Avi-Cys22**


*^∗^Only post-translationally modified residues are displayed. Bolded residues are involved in a lanthionine (Lan, Ala-S-Ala) and/or 3-methyl-lanthionine (MeLan, Abu-S-Ala) bridges. Dha, 2,3-didehydroalanine; Dhb, 2,3-didehydrobutyrine; AviCys, aminovinyl-*D*-cysteine.*

Despite a substantial amount of evidence that demonstrate that lantibiotics are efficacious and that they would be well tolerated in humans (Smith and Hillman, 2008; Ross and Vederas, 2011; Piper et al., 2012; Götz et al., 2014; Field et al., 2015; Ongey et al., 2017), only a few have advanced to clinical trials: NVB333 (methicillin-resistant *Staphylococcus aureus* infections (MRSA) and VRE infections) are currently in pre-clinical testing, NVB302 (*C. difficile* infections) completed Phase-I, and Duramycin (Cystic Fibrosis) completed Phase-II (Sandiford, 2015; Boakes et al., 2016; Ongey et al., 2017).

Mutacin 1140 is a lantibiotic produced by the Gram-positive bacterium *Streptococcus mutans* that was discovered independently by two different groups (Hillman et al., 1984, 1998; Qi et al., 1999). Thirty-three years were spent characterizing, producing and purifying enough of the compound to sufficient quantities with high enough purity to investigate its potential as a novel therapeutic agent. During that period, the polycistronic operon of MU1140 was mapped (Hillman et al., 1998) and entirely sequenced (Escano et al., 2016), its *in vitro* susceptibility profile and spectrum of activity were established (Ghobrial et al., 2009), a low frequency of development of antimicrobial resistance was substantiated (Ghobrial et al., 2009), the low levels of cytotoxicity and good overall pharmacological profile were reported (Ghobrial et al., 2009, 2010; Oragenics, unpublished), and its high-resolution structure defined by nuclear magnetic resonance (NMR) spectroscopy (Smith et al., 2003). Of particular interest, MU1140 was found to exert its antimicrobial activity via a novel mechanism of action termed Lipid-II abduction (Hasper et al., 2006; de Kruijff et al., 2008; Smith et al., 2008), which is less prone to natural development of resistance because of the ancestral nature of the pyrophosphate moiety of Lipid-II (Ghobrial et al., 2009). Lipid II is an essential component of peptidoglycan synthesis that is targeted by several antibiotics, including vancomycin (Brötz et al., 1998). In contrast to vancomycin, which binds to Lipid II at the terminal part of the pentapeptide, MU1140 (and nisin) binds the pyrophosphate moiety of Lipid-II. Following selection by exposure to vancomycin, bacterial species have evolved the ability to change the composition of the pentapeptide of Lipid-II without impeding cell wall synthesis. For instance, VREs that acquire *van*A develop vancomycin-resistance by the substitution of the *D*-Ala-*D*-Ala of the pentapeptide of Lipid-II by a *D*-Ala-*D*-Lactate moiety (Hsu et al., 2004). In contrast, no bacterial species have yet been identified that can effectively modulate the structure of the pyrophosphate moiety of Lipid-II without impacting its function, which makes it an attractive target for the development of antibiotics. Consequently, the critical and ancestral nature of the pyrophosphate moiety of Lipid-II implies that the development of resistance would be infrequent (Ghobrial et al., 2009). The use of nisin in the food industry since 1951 (in over 50 countries) certainly supports that hypothesis.

Mutacin 1140 (see **Figure 1**) is an attractive scaffold for future antibiotic engineering by virtue of its excellent safety profile, novel mechanism of action and consequently its limited potential for development of resistance in the clinic. We have previously proposed a new set of blueprints that may enable and foster future therapeutic development of lantibiotics, taking into account classic pitfalls related to their production, purification and formulation. These issues have historically hindered the “drugability” of MU1140 as well as other lantibiotics. A saturation mutagenesis library was previously created to study the contribution of every amino acids of the core peptide of MU1140, in an unbiased and addressable fashion (Kers et al., unpublished), to create 418 different variants of MU1140 (22 positions × 19 possible permutations). In the current report, we triage the best performers from the saturation mutagenesis library in order to select a lead compound for the treatment of *C. difficile* infections based on: (a) physicochemical properties, (b) *in vitro* susceptibility levels, (c) spectrum of activity, (d) stability and solubility in bio-relevant fluids (FaSSGF, FaSSIF and serum), and (e) efficacy *in vivo*.

## Materials and Methods

### Strains

The construction of the MU1140 variant strain library has been detailed previously (Kers et al., unpublished) see **Table 2**. Briefly, variants were constructed in *S. mutans* JH1140 (Hillman et al., 1984) by allelic replacement where the native chromosomal *lan*A gene was replaced with *lan*A variants encoding codon substitutions. Splicing by Overlap Extension (SOE) PCR was used to construct DNA vectors for integration of *lan*A variants into the JH1140 chromosome using a selectable erythromycin resistance marker (from pVA891, Macrina et al., 1983). Strains were routinely grown on triptic soy broth or agar containing 0.2% yeast extract (TSYEX), 3 μg/mL erythromycin, and incubated in a candle jar for 3 days at 37°C. PCR and Sanger DNA sequencing was utilized to confirm replacement of the chromosomal copy of *lan*A with the *lan*A variant encoded on the integration vector. A high-throughput method was used to confirm the biological activity of each variant strain using *Micrococcus luteus* as a reporter strain (see details in Kers et al., unpublished). Strains are denoted as SMxxx, while their corresponding lantibiotics are labeled as OGxxx (e.g., strain SM253 produces the lantibiotic OG253).

**Table 2:** Selected^1^ strains used in this study.

Name	Property, genotype or characteristics	Source or reference
*S. mutans* JH1140	MU1140 hyperproducing strain of *S. mutans* derived from JH1000	Hillman et al., 1984
*S. mutans* SM152	JH1140::Erm (intergenic *lan*A’-*lan*B) (producing MU1140)	Kers et al., unpublished
*S. mutans* SM126	*S. mutans* JH1000 ΔlanAA′ (*lan*A^-^)	Kers et al. unpublished
*S. mutans* SM157	SM152 producing OG157 (Phe17Leu)	Kers et al., unpublished
*S. mutans* SM161	SM152 producing OG161 (Asn18Ala)	Kers et al., unpublished
*S. mutans* SM168	SM152 producing OG168 (Tyr20Phe)	Kers et al., unpublished
*S. mutans* SM179	SM152 producing OG179 (Phe1Leu)	Kers et al., unpublished
*S. mutans* SM191	SM152 producing OG191 (Arg13Asn)	Kers et al., unpublished
*S. mutans* SM253	SM152 producing OG253 (Phe1Ile)	Kers et al., unpublished
*S. aureus* ATCC 29213	CLSI control, methicillin susceptible	Eurofins
*S. aureus* ATCC 19636	Smith, Osteomyelitis isolate, *in vivo* strain	Eurofins
*S. aureus* ATCC 33591	MRSA, *in vivo* strain	Eurofins
*S. aureus* BAA-1717	Community acquired MRSA USA300 SCCmec type IVa PVL+	Eurofins
*S. aureus* ATCC 700699	Mu50 VISA	Eurofins
*S. pneumonia* ATCC 700675	PEN-R Macrolide-R, South Africa 6B-8	Eurofins
*M. phlei* ATCC 11758	-	Eurofins
*P. aeruginosa* TUH-974	MDR clinical isolate	Eurofins
*C. difficile* ATCC 9689	Ribotype 001	Eurofins
*C. difficile* BAA-1805	Ribotype 027 (NAP1) hypervirulent strain	Eurofins
*C. difficile* BAA-1875	Ribotype 078	Eurofins
*C. difficile* UNT103-1	VA11, non epidemic (cdtB-, REA group J)	UNTHSC^2^
*E. faecalis* ATCC 51575	VRE VanB strain	Eurofins
*E. faecalis* ATCC 51299	VRE VanB strain	Eurofins
*E. faecium* ATCC 700221	VRE VanA strain	Eurofins


*^*1*^Entire *S. mutans* strains SMxxx library can be found in Kers et al., (unpublished). ^*2*^Received from Ohio VA Medical Center (Curtis Donskey).*

### Compound Manufacturing and Characterization by Ultra-High Performance Liquid Chromatography/Mass Spectrometry (UPLC/MS), HPLC and Nuclear Magnetic Resonance

Compounds tested in this study were produced by fermentation and purified by Intrexon Corp. Detailed experimental conditions have been previously reported (Kers et al., unpublished). Larger scale (∼100 mg) manufacturing was carried out at Oragenics using proprietary methods (Oragenics, unpublished). Briefly, MU1140 chromosomal *lan*A variant strains were grown in Sartorius Biostat A plus 1 L bioreactor using fed-batch fermentations under aerobic stirred-tank conditions with automated temperature/pH/dissolved oxygen controls. Culture supernatants were extracted using chloroform extractions to isolate the compounds of interest (modified from Hillman et al., 1984). Purification was carried out by flash chromatography. This scale was adequate to generate sufficient purity and quantities of compounds to perform all assays contained in the current report.

The purity and identity of each variant was determined by UPLC/MS as previously reported (Kers et al., unpublished). The purity of variants during solubility and stability studies was determined by HPLC on a Waters XBridge C18 column, particle size 3.5 μm, 3.0× 150 mm. Buffer A was 0.05% trifluoroacetic acid (TFA) in H_2_O and Buffer B was 0.05% TFA in 5% H_2_O/95% acetonitrile (ACN). The gradient was 2% buffer B to 85% buffer B over 38 min at flow rate of 0.6 mL/min. Injection volume was 100 μL.

NMR spectra of OG253 (MU1140-Phe1Ile) were acquired on an Agilent NMRS instrument, operating at 600 MHz for proton, and equipped with a High Temperature Superconductor (HTS) 1.5 mm probe (University of Florida NMR Core Facility). The water signal was suppressed in a wet 1D experiment. The precedent parameters were used to setup: (a) a Total Correlation Spectroscopy (TOCSY) experiment with a mixing time of 150 ms, (b) a Rotating-Frame NOE Spectroscopy (ROESY) experiment with a mixing time of 200 ms, (c) a Heteronuclear Single Quantum Coherence (HSQC) experiment optimized for a one-bond coupling constant of 146 Hz and (d) a Heteronuclear Multiple Bond Correlation (HMBC) experiment optimized for a long-range coupling constant of 8 Hz. A 5 mg sample of OG253 was dissolved in 60 μL of a mixture of deuterated acetonitrile:water, 3:1, yielding a 37 mM solution (Smith et al., 2000).

### Minimum Inhibitory Concentration (MIC) Testing

MIC testing was performed by Eurofins Panlabs Taiwan LTD. Briefly, MICs were measured using the broth dilution assay as described by the CLSI for aerobic bacteria (CLSI, 2009), and a modification of the CLSI M11-A8 broth dilution method for *Bacteroides fragilis* susceptibility testing was used for *C. difficile* (CLSI, 2012). The strains tested are presented in **Table 2**. Briefly, lyophilized compounds were dissolved and diluted with 100% dimethyl sulfoxide (DMSO). All compounds were tested with a standard reference agent for each strain (ampicillin, gentamicin, vancomycin, ofloxacin, or linezolid). Each test article or reference agent were tested at 11 concentrations by twofold serial dilutions from 64 to 0.0625 μg/mL, incubated and then visually examined and scored positive (+) for inhibition of growth or turbidity or negative (-) for no effect upon growth or turbidity. The final concentration of DMSO was 2%. Each concentration was evaluated in duplicate. The MIC was defined as the lowest concentration of an antimicrobial agent that completely prevented visible growth of a microorganism in the broth susceptibility test. Vehicle-control and active reference agents were used as blank and positive controls, respectively. The MIC values of ampicillin, gentamicin, vancomycin and ofloxacin controls were consistent with the historical data against test strains (data not shown).

### *In Vitro* Cytotoxicity

The HepG2 cytotoxicity evaluation was performed by MB Research Labs (Spinnerstown, PA, United States) by the Neutral Red Uptake Bioassay of HepG2 human hepatocellular carcinoma cells in culture (ATCC #HB-8065). Briefly, HepG2 cells were seeded in the central 60 wells of 96-well plates and maintained in culture for approximately 24 h. Following pre-incubation, the culture medium was replaced with culture medium containing increasing concentrations of the test articles or control in the vehicle. Test articles were tested in triplicate wells, and reported as the mean. The cells were incubated for 24 h and cell viability was determined by Neutral Red (NR) uptake. For this assay, the medium was then replaced with NR medium (50 μg/mL NR in Hanks’ Balance Salt Solution, HBSS) and the cells were incubated for an additional 3 h. Following uptake of NR, the medium was discarded, the wells were washed with HBSS, and lysing solution (50:49:1 ethanol:water:acetic acid) was added subsequently to each well. The plates were placed on a rocker for 30 min until NR had been extracted from the cells and formed a homogeneous solution. The absorbance of each well was measured at 540 nm on a μQuant^TM^ plate reader (Bio-Tek Instruments) using KCjunior^TM^ software. The mean of the outer wells (DMEM2-FBS) was used as a reference control. The mean percent viability was determined for each concentration compared to the vehicle-treated control. A positive result was determined by: (1) a >25% decrease in absorbance of test article-treated cells calculated as a percentage of vehicle-treated cells, and/or (2) *p* < 0.05 of test article-treated cells using the Student’s *t*-test. Where possible, the concentration at which cell viability was reduced to 50% (EC_50_) was calculated.

### *In Vitro* Safety Screen 44^TM^ Pharmacologic Profiling

The *in vitro* pharmacology profiling was performed by CEREP Labs (now Eurofins Panlabs, Celle-Lévescault, France). Compounds were tested in Safety Screen 44^TM^ panel, which is for off-target profiling using a compilation of classical competition binding assays and enzymatic inhibition assays performed with human recombinant proteins (Bowes et al., 2012). Experimental parameters and conditions for each of these 44 assays were as per the manufacturer’s testing information^1^. The data reported is expressed as the mean of duplicate experiments. The respective reference compound was tested concurrently with the test compounds, and the data were compared with historical values determined at Cerep. The experiment was accepted in accordance with Cerep’s validation Standard Operating Procedure. The Safety Screen 44^TM^ results showing an inhibition (or stimulation for assays run in basal conditions) higher than 50% are considered to represent significant effects of the test compounds. 50% is the most common cut-off value for further investigation (determination of IC_50_ or EC_50_ values from concentration-response curves) that the manufacturer recommends, based on historical data. Results showing an inhibition (or stimulation) between 25 and 50% are indicative of weak to moderate effects (in most assays, they should be confirmed by further testing as they are within a range where more inter-experimental variability can occur). Results showing an inhibition (or stimulation) lower than 25% are considered insignificant and mostly attributable to variability of the signal around the control level. High negative values (≥50%) that are sometimes obtained with high concentrations of test compounds are generally attributable to non-specific effects of the test compounds in the assays. On rare occasions they could suggest an allosteric effect of the test compound.

### Metabolic Stability in Fasted State Simulated Gastric Fluid (FaSSGF), Fasted State Simulated Intestinal Fluids (FaSSIF) and Serum

The studies highlighted below were performed by Seventh Wave Laboratories and the University of Massachusetts, Lowell (see **Table 3**). Fasted State Simulated Gastric Fluid (FaSSGF) was prepared with 2.0 g NaCl in 7.0 mL conc. HCl and sufficient water (QS) to make 1000 mL (final pH 1.5), with and without 3.2 g purified porcine pepsin (800 – 2500 U/mg, Fisher Scientific). Pepsin is the major proteolytic enzyme produced in the stomach and it is known to cleave peptides with an aromatic acid on either side of the peptide bond (Fruton, 2002). A stock of each of the compounds for testing was prepared at 10 mg/mL in 10% ACN. Initially, 242.5 μL of FaSSGF ± pepsin was added to snap-lid 1.5 mL Eppendorf tubes. To each tube, 7.5 μL of compound at 10 mg/mL was added for a final concentration of 300 μg/mL. All tubes were incubated at 37°C. At each time point, a 40 μL aliquot was removed from each tube, and the protease activity in the reaction aliquot was inactivated by addition of 1 μL of formic acid and 120 μL of ACN. Time points collected were at 0, 0.5, 1, 2, and 4 h. For negative control, only 0 and 4 h time points were collected. Post-precipitated samples were centrifuged at 20,000 × *g* for 10 min in a table top centrifuge at room temperature. A 75 μL portion of the supernatant was transferred to a HPLC vial and 450 μL of 0.05% TFA in water was added for HPLC analysis (see HPLC Method above).

**Table 3:** Metabolic and chemical stability, solubility and half life in biologically relevant fluids.

	30% HPβCD		5% Mannitol		Half-life		
			
Compound^1^	Solubility^2^	Stability^2,3^	Solubility^4^	Stability^3,4^	FaSSGF^4^	FaSSIF^5^	Serum^5^
	(mg/ml)	(%)	(mg/ml)	(%)	(min)	(min)	(min)
MU1140	<8	-7.6	41.0	-0.2	>240	72.4	107
OG157 (Phe17Leu)	<8	-3.2	166.5	0.4	ND	4.9	37.1
OG161 (Asn18Ala)	<8	0.3	181.6	-0.1	>240	<30	<60
OG168 (Tyr20Phe)	<8	-0.4	41.5	-0.3	>240	148	195
OG179 (Phe1Leu)	<8	-2.0	161.6	0.4	>240	77.3	60.8
OG191 (Arg13Asn)	29	6.0	39.7	-0.9	>240	>240	>107
OG253 (Phe1Ile)	<8	-13.4	169.2	0.2	>240^6^	95.0	270


*^*1*^Substitutions from MU1140 in parenthesis. ^*2*^Performed at Almac Group. ^*3*^Stability expressed as the difference of purity pre- versus post-incubation. Incubation at room temperature, 72 h. ^*4*^Performed at UMASS Lowell. Data reported as the mean of triplicate experiments. ^*5*^Performed at Seventh Wave Laboratory, with confirmations at UMASS Lowell. Data reported as the mean of triplicate experiments. ^*6*^Confirmed at Oragenics.*

Fasted State Simulated Intestinal Fluid (FaSSIF) was prepared as follows: FaSSIF-v2^2^ solution in maleate buffer which has the composition of 3 mM sodium taurocholate, 0.2 mM lecithin, 19.12 mM maleic acid, 34.8 mM NaOH, 68.62 mM NaCl (final pH 6.5), with and without protease (2% Pancreatin (w/v), 6 μg/mL of trypsin and 6 μg/mL of α-chymotrypsin (Foltmann, 1981). All proteases were from Fisher Scientific. A stock of each compound under investigation was prepared at 10 mg/mL in 10% ACN. Initially, 7.5 μL samples of compound at 10 mg/mL were added to 1.5 mL Eppendorf tubes, and 242.5 μL of FaSSIF ± enzymes were added to each sample (final concentration of compound of 300 μg/ml). Samples were incubated at 37°C in closed tubes. Time points collected were at 0, 0.5, 1, 2, and 4 h. For FaSSIF without enzymes (negative control), only 0 and 4 h time points were collected. At each time point, a 40 μL aliquot was taken from each tube. The protease activity in the aliquot was stopped and the proteins precipitated with 1 μL of formic acid and 120 μL of ACN. Precipitated samples were then centrifuge at 20,000 × *g* for 10 min in a table top centrifuge at room temperature. A 75 μL volume of supernatant was transferred to an HPLC vial and 450 μL of 0.05% TFA in water was added for HPLC analysis using the previously described method.

Stability in human serum was tested as follows: a 10 mg/mL solution of each compound was made in 10% ACN. A 7.5 μL volume of the 10 mg/mL compound stock was added to 242.5 μL of 100 mM acetate buffer at (pH 5.5) in a 1.5 mL Eppendorf tube (for a final concentration of). A 242.5 μL volume of complement preserved serum (0.2 μm filtered, pooled gender, Bioreclamation, Cat# HMSRM-COMP) was added to the tube (final concentration of 300 μg/mL per compound). Negative controls did not contain serum. To both the serum tube and the negative tube, samples were incubated covered at 37°C. Time points collected were 0, 0.5, 1, 2, 4, and 8 h. For negative control, only the time 0 and 8 h time points were collected. At each time point, a 40 μL aliquot was taken from each tube for HPLC analysis (see Materials and Methods).

All metabolic stability FaSSGF, FaSSIF and serum data is reported as the mean of triplicate experiments.

### Efficacy Assessment *in Vivo*

#### Animals and Test Organism

Male Golden Syrian hamsters were used in this study weighing 80 – 90 g (Charles River Laboratories, Wilmington, MA, United States), housed one per cage with free access to food and water, in accordance with NIH guidelines. *C. difficile* UNT103-1 (VA11, non-epidemic (cdtB-, REA group J) was received from Curtis Donskey, Cleveland VA Hospital, Cleveland, OH, United States). The isolate has been previously utilized for the hamster model (Weiss et al., 2014) and is part of the University of North Texas Health Science Center culture collection and was preserved in a Trypic Soy Broth stock containing 20% glycerol at -70°C. This study was carried out in accordance with protocols 2012/13-14-A06, 2016-0019, and 2016-0015 approved by the Institutional Animal Care and Use Committee (IACUC) at the University of North Texas Health Science Center (UNTHSC). IACUC established guidelines ensuring that approved protocols are in compliance with federal and state laws regarding animal care and use activity at UNTHSC. The UNTHSC animal program is USDA registered (74-R0081) and fully AAALAC accredited.

#### Surgical Procedure

On the day of surgery, each animal was anesthetized with 3–4% isoflurane and a midline incision generated through the ventral peritoneum. While retracting the incision, the ileum proximal to the ileocecal junction was isolated and temporarily ligated with 2 – 0 silk suture. A 1 – 2 mm transverse incision was made within the ligated ileal section and the tippet of an MRE-40 catheter (Braintree Scientific) inserted through the incision and advanced into the ileum to a length of ∼1 cm toward the ileocecal junction. The catheter was anchored to the ileum with 5 – 0 monofilament suture, and the cannulated ileum placed back into the peritoneal cavity. The peritoneal incision was then closed and the remainder of the catheter routed subcutaneously (SC) for external porting through the skin near the scapular region. Animals were given 2 mg/Kg meloxicam as needed for pain and discomfort. Animals recovered for a period of 10 days after surgery before infection.

#### Surgical Days and Infection

A total of 60 animals were implanted with ileal cannulas on 3 – 4 separate days (to achieve 48 animals for study), as presented in **Table 4**. Animals from all days were randomized into the 8 study groups (*N* = 6 per group) prior to treatment. UNT103-1 was passaged onto trypticase soy agar supplemented with 5% sheep blood (TSA + SB) plates 4 days before infection and anaerobically incubated in an anaerobic chamber at 37°C for 48 h. After incubation, the plate growth was suspended into 20 mL of pre-reduced tryptone-glucose-yeast extract (TGY) nutrient broth and anaerobically incubated at 37°C for 24 h. At 24 h, the 24 h culture was diluted 10-fold into SM sporulation medium (SM broth, Wilson et al., 1982) and anaerobically incubated at 37°C for 48 h. On the day of infection (day 1, 48 h post-inoculation of SM broth), the OD of the SM broth culture was adjusted to an absorbance (OD600nm) of 1 (∼1.0E+09 CFU/mL) in pre-reduced SM broth, and hamsters were orally infected with 0.5 mL of the ∼1.0E+09 CFU/mL (∼5.0E+08 CFU), and the OD adjusted suspension was 10-fold serially diluted in pre-reduced TGY and spot plate onto TSA + SB (5%) to confirm the input CFU per animal. The OD-adjusted suspension (∼1.0E + 09 CFU/mL) was ethanol shocked by diluting 0.2 mL of the suspension into 0.2 mL of ethanol and the mixture was incubated at room temperature for 60 min. The mixture was cold-centrifuged (4°C) for 5 min at 10,000 × *g*. The supernatant was decanted and the cellular/spore pellet was suspend in 0.4 mL of sterile 1X PBS and centrifuged as previously described. The supernatant was decanted and the cellular/spore pellet was suspended in 0.2 mL of sterile 1X PBS. The ethanol shocked mixture was 10-fold serially diluted in pre-reduced TGY and spot plated onto TSA+SB (5%) to determine percentage of spores relative to vegetative cells.

**Table 4:** cHCDAD study design.

Group	Test article(s)	Regimen^a^	Route	Dose (mg/Kg)	*n*
1	OG157 (Phe17Leu)	TID × 5 days	Ileal canula	20	6
2	OG253 (Phe1Ile)	TID × 5 days		20	6
3	OG168 (Tyr20Phe)	TID × 5 days		20	6
4	OG179 (Phe1Leu)	TID × 5 days		20	6
5	OG161 (Asn18Ala)	TID × 5 days		20	6
6	OG191 (Arg13Asn)	TID × 5 days		20	6
7	Vancomycin	QD × 5 days		20	6
8	Infection (Vehicle) Control	TID × 5 days		NA	6


*^*a*^Animals were infected with spores on day 1, treated with clindamycin on day 2 (at 24 h after infection), and formulations were administered starting on day 3 (18 h after clindamycin injection) for 5 consecutive days (days 3 through 7). Animals were observed until the end of the relapse period, through day 21. See Section “Materials and Methods” for additional details.*

#### Clindamycin and Treatment

On day 2, at 24 h after infection, all animals received a single subcutaneous injection of clindamycin (10 mg/Kg). Variant formulations were administered at 20 mg/Kg, three times a day (TID), starting on day 3 (18 h after clindamycin injection) for 5 consecutive days (days 3 through 7) through the surgically implanted ileal canula. Vancomycin (positive control) was prepared in water for injection (WFI) at 50 mg/mL and stored at 4°C over the course of the study. Variant formulations and vancomycin were diluted fresh each day to the appropriate concentration and administered at 20 mg/Kg on days 3 through 7 via the canula and the infection control group was dosed with Test Article vehicle in the same manner. All Test Articles was dosed with ≤0.2 mL volume followed by a 0.1 mL WFI rinse.

#### Study Design

The design of this study is detailed in **Table 4**. All Test Articles and Vehicle Control were dosed with 0.19 mL volume followed by a 0.1 mL WFI rinse. The identity and concentration of test articles were kept blinded during the study, and 5 individual aliquots of 4.1 mL/tube were provided frozen at ≤-70°C. Each tube, containing sufficient volume for one full day of dosing 6 animals TID with ∼20% excess, was thawed at the morning dose and kept refrigerated (5°C) between doses. Following the last dose of the day, the remaining solution was returned to ≤-80°C storage. Following completion of the study, all tubes containing test articles were returned to Oragenics for testing, to confirm stability, purity and activity (data not shown).

#### Monitoring

Animals were observed a minimum of three times a day for the duration of the experiment, and more often as appropriate based on clinical condition. General observations included signs for mortality and morbidity, for the presence of diarrhea (“wet tail”), overall appearance (activity, general response to handling, touch, ruffled fur) and were recorded. Body weights were measured and recorded every other day. At the same time, temperatures were measured using a hand-held infrared thermometer for each animal and also recorded. Animals judged to be in a moribund state were euthanized by CO_2_ inhalation. Criteria used to assign a moribund state were extended periods (5 days) of weight loss, or progression to an emaciated state, or prolonged lethargy (more than 3 days), or signs of paralysis, or skin erosions or trauma, or hunched posture and a distended abdomen. Observations were continued, with any deaths or euthanasia recorded, for a period up to 21 days post-infection in order to identify animals suffering a relapse of *C. difficile* infection.

#### Endpoint and Collected Samples

The cecal contents from all hamsters that died during the study, or from those hamsters euthanized by CO_2_ inhalation at the end of the observation period (day 21), were collected and aliquoted into two samples. One sample was used for *C. difficile* TOX AB test (tgcBIOMICS GmbH, Bingen, Germany) and the other stored frozen at -80°C for use in total (spore and CFU) viable counts.

Samples were collected by isolating the cecum through an incision made in the right peritoneal (abdominal) region. The ileocecal junction was carefully exposed by removing the surrounding mesentery and adipose tissue. The ileocecal section of each animal was observed, recorded and photographed. Using minimally serrated forceps, the cecum was picked up so that the end was positioned above the animal. The cecal wall was carefully punctured immediately placed into an open 14-mL Falcon tube. After the contents drained into the tube, the cecal contents were diluted with an equal volume of sterile 1xPBS. The ileum and cecum were removed and stored and tested for CFU/spores as presented above.

#### Data Analysis

Data was analyzed using GraphPad Prism 6.0d. Survival was compared using Logrank test and Gehan-Breslow-Wilcoxon test. Statistical significance was set at *p* < 0.05.

## Results

### Compound Selection Strategy

**FIGURE 2:**
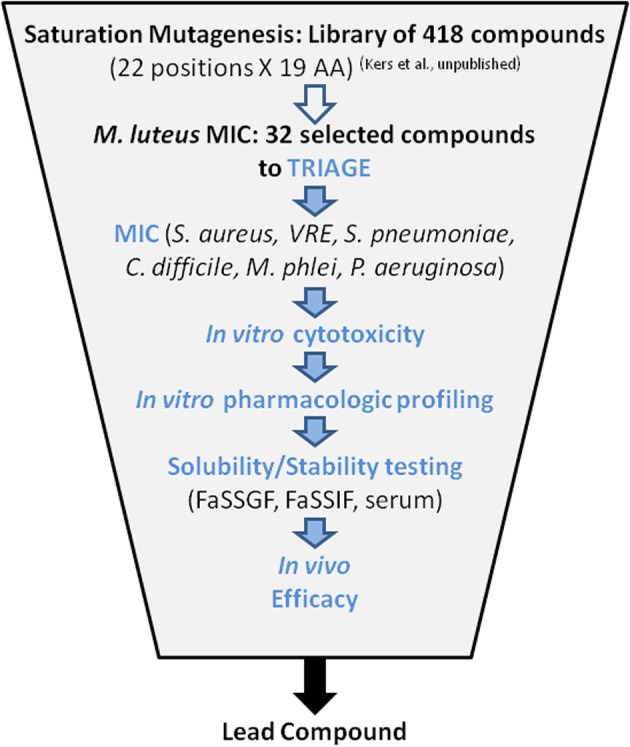
Triage strategy.

Thirty-two compounds were selected from a saturation mutagenesis library of 418 single amino acid variants of MU1140 (Kers et al., unpublished). An average of 19.5 mg (0.67 mg – 47.1 mg) of each compound was isolated and purified, to >90% purity by flash chromatography (Kers et al., unpublished). Each compound was subjected to a battery of tests designed to identify a reasonable number of lead compounds for animal testing. The overall triage strategy is illustrated in **Figure 2** which included (a) *in vitro* susceptibility assay, (b) *in vitro* toxicity assessment, (c) characterization of chemical stability, and (d) characterization of metabolic stability. The top 6 overall performers from the *in vitro* assays were ultimately tested to evaluate their *in vivo* efficacy, in order to ultimately select a lead compound.

### Characterization

The identity of purified compounds was characterized by LC/MS. The dominant peak of 31 compounds confirmed the expected mass (**Supplementary Table S1**). However, Leu6His was characterized by a 23 Da lower mass than expected (calculated mass: 2288.62 Da; observed mass: 2264.8 Da). The cause of the apparent 23 Da mass difference from the expected value is not known. Minor product peaks that appeared common to several variants were identified as the deletion of Phe1. Ten out of 32 variants had a Phe1 deletion. Minor products were also observed with several variants, such as oxidation (+16 Da) and dehydration (-18 Da). It was speculated that the sulfur atom in Ser5Met was oxidized (+16 Da) during manufacturing. The loss of 18 Da would support the concept that the Thr from Leu6Thr and Arg13Thr underwent dehydration (-18 Da) during post-translational modification to give Leu6Dhb and Arg13Dhb, respectively (Kers et al., unpublished). The lead compound (OG253, aka MU1140-Phe-1Ile) was further characterized to confirm its primary sequence by extended NMR determination (Smith et al., 2000).

### *In Vitro* Susceptibility Profile

To assess the *in vitro* susceptibility profile and characterize the spectrum of activity of selected variants of MU1140, the MIC was tested against a small panel of representative Gram positive bacterial pathogens. The species selected included *C. difficile* (*n* = 3), VRE (*n* = 3), *Staphylococcus aureus* (*n* = 5), *Streptococcus pneumonia* (*n* = 1) and *Mycobacterium phlei* (as s surrogate for *M. tuberculosis*, *n* = 1). *Pseudomonas aeruginosa* (*n* = 1) was also selected as an example for Gram negative organisms. *M. luteus* was used as a control to monitor consistency with previous reports. The complete MIC dataset is presented in **Supplementary Table S1**. Because of the small sample size used with all other bacterial species, only the MIC data on *S. aureus* allowed for meaningful mode and range calculation. Some degree of variability between strains was observed. In general though there was good consistency in the detected activity of a compound across the panel of Gram-positive organisms tested; a compound that was relatively potent with *M. luteus* was also relatively potent with other Gram positive species tested, with a few exceptions. In contrast, all compounds were equally ineffective against *P. aeruginosa*. Several compounds with adequate MIC against one or several bacterial species were identified and prioritized in subsequent testing. In particular, at least seventeen (17) compounds were identified with equal or superior *in vitro* susceptibility profiles against *C. difficile*, compared to vancomycin (range 0.5 – 4): Phe1Ile, Phe1Leu, Phe1Tyr, Lys2Ile, Lys2Met, Ser5Met, Leu6His, Leu6Val, Arg13Ala, Arg13Leu, Arg13Asn, Arg13Thr, Gly15Ser, Phe17Leu, Phe17Tyr, Asn18Ala, and Tyr20Phe. One compound (Lys2Met) was identified with a superior i*n vitro* susceptibility profile against VRE, compared to Linezoid, but the significance of this data point was questioned considering the relatively low amounts and mass accuracy of material available for testing (0.67 mg). A few compounds were isolated with similar range as vancomycin against *S. aureus* (0.125 – 2), including Phe1Ala, Leu6His, Phe17Tyr, and Tyr20Phe. Not a single compound was identified with equal or superior MIC to *S. pneumoniae* as compared to ampicillin (MIC = 1.0). The *in vitro* susceptibility profile of *M. phlei* ranged 2 – 16 overall and was inferior to vancomycin (MIC = 0.25). Many compounds that were relatively potent with *M. luteus* demonstrated a lower relative *in vitro* susceptibility profile with other species. While this may have served as a rationale basis for deprioritization, their further characterization was still deemed useful and pursued to investigate whether their MIC could be correlated with other characteristics.

### *In Vitro* Cytotoxicity

A monolayer of a human immortalized liver cell line (HepG2) was used as a first-pass, high-throughput, and inexpensive system to assess potential overt signs of cellular toxicity. Cytotoxicity was assessed using a neutral red uptake bioassay (MB Research Laboratories). The mean percentage viability was determined for each compound tested and compared to vehicle-treated control. A positive result was determined by a greater than 25% decrease in percentage of vehicle-treated cells. When possible, the concentration at which cell viability was reduced to 50% (EC_50_) was calculated. The complete dataset is presented in **Supplementary Table S1**. The vehicle-alone formulation did not negatively impact the viability of HepG2 cells (data not shown). Interestingly, cytotoxicity was undetectable (maximum concentration tested = 1,000 μg/mL) for approximately half of the compound tested (14 of 32 compounds, or 44%). The remainder had variable levels of toxicity reflected by their EC_50_ between 171 μg/mL and 805 μg/mL (μM range). The overall range of cytotoxicity remains approximately 3 orders of magnitude higher than the expected therapeutic range (low μM to high nM).

### *In Vitro* Pharmacological Profiling

The complete *in vitro* pharmacology profiling dataset as investigated with the Safety Screen 44^TM^ is presented in **Supplementary Table S1**. Altogether, the results showed no consistent target hits in this library of compounds. The profiles were more indicative of low level of random, non-specific inhibition, based on unique structural attributes of individual lantibiotic variants. The data indicate that this class of molecules has limited off-target interactions based on their pharmacological profiles. The data also suggests that no single amino acid variants of MU1140 presents with a drastically different pharmacologic profile relative to MU1140. Mutacin 1140 was positive for only 3 of the 44 assays, each falling within a different functional family. The positive hits included a G protein-coupled receptor (GPCR, delta2 DOP), an ion channel receptor (NMDA) and a kinase (CTK). The amplitude of the signal obtained 42, 25, and 47% respectively, indicative of weak to moderate effects for all targets. Those targets were also found positive with several other variants besides MU1140 (see **Supplementary Table S1** for details). A total of 15 of the 44 targets returned a hit for all MU1140 variants tested (all levels of signal considered). Promiscuity analysis enabled benchmarking of the overall pharmacology profile of MU1140 variants against a library of known compounds (Bowes et al., 2012). Defining the target hit rate as the percentage of targets with >50% inhibition (**Supplementary Table S1**) demonstrate that all MU1140 variants showed a low level of promiscuity (between 0 and 5% of targets are considered selective). It is noteworthy to mention that all high impact targets tested demonstrated a low hit frequency. For example, Ser5Ala was the only variant with a positive hit for hERG (23%, insignificant); the muscarinic acetylcholine receptor M1 had no positive hits; the cyclooxygenase Cox-1 and Cox-2 only had 9 and 1 positive hits, respectively, but they were all considered insignificant (<25% inhibition). The potential correlation between cytotoxicity on HepG2 cells and the *in vitro* pharmacology profiling was investigated, but failed to reveal a positive or negative correlation (data not shown).

### Metabolic Stability

Therapeutic peptides are inherently unstable and prone to both chemical and metabolic degradation. The physicochemical environment that a therapeutic peptide encounters depends on the route of administration. The inherent chemical/metabolic stability was characterized for a subset of compounds: (1) by degradation analysis, (2) in two biologically relevant models that simulate the gastrointestinal (GI) environment, and (3) in serum. The contribution of proteolytic degradation was also tested in the biologically relevant simulated fluids by addition of a cocktail of enzymes found in the GI tract. The subset of compounds selected represented compounds with similar *in vitro* susceptibility profiles, cytotoxicity and pharmacological profiles, but anticipated to likely show different stability profiles to chemical and/or proteolytic degradation based on key structural differences. **Table 3** summarizes the stability data that was obtained from these studies. The stability in fasted state simulated gastric fluid (FaSSGF) was consistently high for all compounds tested. In contrast, the stability in fasted state simulated intestinal fluid (FaSSIF) was highly variable amongst the compounds tested, reaching low levels with certain compounds (e.g., Phe17Leu was almost entirely degraded at the completion of this assay). Interestingly, several compounds were identified that presented a better FaSSIF profile than MU1140, suggesting that their resistance to proteolytic degradation could be improved in the GI tract by a single amino acid substitution (e.g., Phe1Ile, Arg13Asn and Tyr20Phe). The stability profile in serum paralleled the profile in FaSSIF.

### Efficacy Assessment *in Vivo*

**FIGURE 3:**
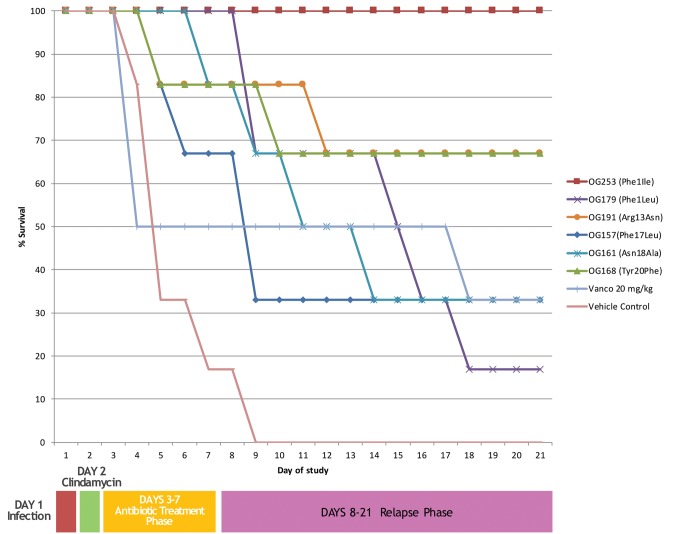
*In vivo* efficacy of top performers. Animals were infected on day 1 followed by clindamycin treatment on day 2. Antibiotics treatment was from days 3–7 and relapse was monitored from until day 21.

The top 6 selected compounds were tested in the cannulated Syrian hamster efficacy/relapse model of *C. difficile* infection (cHCDAD). Efficacy was tested in a cannulated model to minimize the potential premature proteolytic or acid hydrolysis-based degradation in the upper GI tract and the results are presented in **Figure 3**. The vehicle controls demonstrated 100% mortality by day 9, while vancomycin-treated animals showed 33% survival at the end of the study period on day 21. In contrast, the lead compound (MU1140-Phe1Ile, aka OG253) showed 100% survival (*p* = 0.0005 versus vehicle control), following 5 days TID dosing. The other selected compounds displayed varying levels of efficacy ranging from 17% – 67% survival. Tyr20Phe and Arg13Asn exhibited comparable efficacy with 67% survival each by the end of the study on day 21. The remaining test articles, Phe17Leu, Phe1Leu and Asn18Ala, were slightly less effective with 33 – 67% survival on day 9 and 17 – 33% survival for all 3 test articles by day 21. Statistical analysis [Logrank (Mantel-Cox) and Gehan-Breslow-Wilcoxon] of the Kaplan–Meier plots for survival of test article versus vehicle controls groups indicated statistical significance (*p* < 0.05) for all MU1140 variants, except for the Phe17Leu variant (Logrank *p* = 0.0523, Wilcoxon *p* = 0.0553). The small number of animal used to support efficacy does not allow for a statistically siginficant effect of relapse to be substantiated when compounds were compared to untreated (*n* = 1 at day 8) or vancomycin-treated animals (*n* = 3 at day 8), where only one of the animal relapsed after the treatment phase (after day 8) for each of the control groups. It is noteworthy to mention that relapse in OG253-treated animals was not observed and that a statistically significant effect on relapse was found for OG253-treated animals (*n* = 6 at day 8) when compared to OG161-treated animals (*n* = 5 at day 8, Logrank *p* = 0.0315, Wilcoxon *p* = 0.0339) or compared to OG179-treated animals (*n* = 6 at day 8, Logrank *p* = 0.0041, Wilcoxon *p* = 0.0061). Post-study analysis of the cecum contents of OG253-treated animals found no detectable *C. difficile* spores (≤1.62Log CFU/g) or Toxin A or B (≤0.27 ng/g) compared to appreciably higher levels detected in vehicle controls (4.09 log CFU, 1061 ng/g Toxin A and 848 ng/g B) and in morbid hamsters (see **Supplementary Figures S1**, **S2**).

## Discussion

The compounds selected in the present study were previously engineered via saturation mutagenesis (Kers et al., unpublished). From the initial library of 418 compounds generated, 32 presented with MICs superior to or in the same range as MU1140 against the sensitive indicator strain *Micrococcus luteus*. The current report further characterizes a subset of those 32 top performing compounds, with the ultimate goal of identifying lead compounds for subsequent clinical testing. The triage strategy was designed to be inclusive of desirable attributes of an antimicrobial such as a high *in vitro* susceptibility (low MIC), a low toxicity profile and adequate physicochemical properties (stability and solubility). There are arguably several approaches that can be utilized to fulfill this objective, but the approach favored in this report took advantage of high-throughput screening that could be performed at a relatively low cost with a large amount of pertinent information useful for identifying a manageable number of leads for animal studies.

Considering the intrinsic MIC profile of MU1140 (Ghobrial et al., 2009), finding a variant with similar MIC is desirable, but not necessarily critical to assure an appropriate therapeutic profile, provided other important pharmacological criteria are met. The panel of human bacterial pathogens selected was by no means exhaustive, but selected based on the high incidence, prevalence, morbidity and/or mortality of the associated diseases, as well as previously reported spectrum of activity and *in vitro* susceptibility profile of MU1140 (Ghobrial et al., 2009). The initial selection of *M. luteus* proved adequate for initial screening of MU1140 compounds as it was confirmed that most compounds that were relatively potent against *M. luteus* were also relatively potent against other Gram-positive species tested. In general, the MICs were consistent with previous reports on MU1140 (Ghobrial et al., 2009) and relatively similar within Gram-positive organisms, except that the MIC of several lantibiotic compounds against *C. difficile* appeared superior compared to other bacterial species, based on the limited number of strains tested. The MIC data on *P. aeruginosa* was consistent with previous reports on MU1140 (Ghobrial et al., 2009) and continue to support the spectrum of activity of MU1140 and its variant compounds against Gram positive organisms. A few compounds were identified with superior *in vitro* susceptibility profiles against VREs and/or *S.* aureus, compared to MU1140, linezolid and vancomycin. In contrast, no compounds were identified with equal or superior MIC as compared to MU1140, ampicillin or vancomycin against *S. pneumoniae* or *M. phlei*. Of particular interest was the identification of at least 17 compounds with equal or superior *in vitro* susceptibility profiles as compared to MU1140 and vancomycin against *C. difficile*. While the large number of potential lead candidates against *C. difficile* may have served as a rationale basis for prioritization of this subset of compounds against *C. difficile* associated diseases (CDAD), additional characterization of all 32 compounds was pursued in order to investigate whether their MIC profile could be correlated with other pharmacological or physicochemical properties.

Hepatotoxicity is the most common reason cited for withdrawal of an approved drug (Xu et al., 2004). Unfortunately, there is no simple solution that exists to adequately predict such adverse events *in vitro* (Xu et al., 2004). The limitations of HepG2 cells were particularly well illustrated in a retrospective study of marketed pharmaceuticals (Xu et al., 2004). The data collected to assess cytotoxicity on an immortalized hepatic cell line suggest that MU1140-related compounds are not significantly cytotoxic, or that this assay is not sufficiently sensitive to detect hepatotoxicity. The low levels of toxicity of MU1140 have previously been observed on different cell lines (Oragenics, unpublished) so the former conclusion appears most logical. It is noteworthy to mention that the low levels of cytotoxicity that were observed at high concentrations with a few compounds which remains approximately 3 orders of magnitude higher than the expected therapeutic concentration.

Recognizing the limitations of *in vitro* cytotoxicity assays in general, the Safety Screen 44^TM^ was used to gather insight on potential off-target pharmacological toxicity of selected compounds. This panel allows the gathering as much *in vitro* pharmacologic toxicity information as possible, in a cost-effective panel that brings together both robustness (each assay is HTS-compatible) and the strategic choice of information-rich targets. This system uses a collection of classical competition binding assays and enzymatic inhibition assays with human recombinant proteins for off-target profiling, aiming at identifying undesirable off-target activities that could potentially hinder or halt the clinical development of a compound. The targets chosen in the Safety Screen 44^TM^ fall into 5 functional classes: G protein-coupled receptors (GPCRs), ion channels, enzymes, transporters and nuclear receptors. This profiling panel has been successfully used by leading pharmaceutical companies and provides early identification of significant off-target interactions for the optimization of safety margins (Bowes et al., 2012). While it is evident that the Safety Screen 44^TM^ is a rational early step in the drug discovery process, it was surprising to note that the current report is the first one, to our knowledge, to take advantage of this tool to characterize lanthipeptides in order to assess their “drugability.” Promiscuity analysis of the 44 targets tested in the Safety Screen 44^TM^ failed to identify consistently strong, important or obvious patterns, based on variations of MU1140 structure. A direct correlation between promiscuity scores and propensity for toxicity is well documented (Bowes et al., 2012) and also applies to high impact target tested such as hERG, the muscarinic acetylcholine receptor M1 and the cyclooxygenases Cox-1 and Cox-2. That a consistent effect of several MU1140 variants on a receptor system was not found provides no immediate concerns for the class that would provide directional information to collect toxicity data in a specific body system. The data does, however, provide some degree of confidence to assess pharmacotoxicity *in vivo* without pre-identified interactions of concern. Interestingly, a weak to moderate effects were observed with MU1140 and a few variants, including a GPCR, delta2 DOP, an ion channel receptor (NMDA) and a kinase (CTK). The potential pharmacological consequence of the interaction of MU1140 with these receptors is unclear, but would only be expected to have a potential toxicological effect in instances where MU1140 came in direct contact with tissues of the nervous system or cells of the immune system, and at the anticipated therapeutic dose. Although of low risk, and largely depending on the intended indication and route of administration, further investigation of those specific off-target interaction may be granted in animals and during first-in-human clinical studies. Altogether, the cytotoxicity and pharmacological screens support the concept that MU1140-derived compounds have low toxicity and limited (if any) off-target interactions when administered orally. The data also suggests that no single amino-acid variants of MU1140 presents with an overall different pharmacologic profile, as compared to MU1140.

As a new chemical entity progresses toward the clinic, its stability characteristics increase in importance. On one hand, an antibiotic is expected to meet strict chemical stability criteria during manufacturing and storage, throughout clinical testing and commercialization. On the other hand, the metabolic stability of a compound can directly impact its PK/PD and overall efficacy. Because peptides are generally recognized as being relatively less stable compared to small molecules, we included this aspect in our testing matrix in an effort to identify variants of MU1140 that would present a desirable stability profile without negatively impacting the MIC of the compound. Peptides are generally sensitive to oxygen, moisture, and pH extremes. They tend to form side products by oxidation, deamidaton, dehydration, hydrolysis and β-elimination (Vlasak and Ionescu, 2011). Based on our LC/MS data, low levels of oxidation were observed with Ser5Met. The acid-stability profile of MU1140 and its variants was consistent with previous reports and anticipated (Ghobrial et al., 2009). In contrast, the stability in simulated intestinal fluids (FaSSIF) was highly variable amongst the compounds tested, reaching high levels of degradation for several compounds. Nevertheless, several compounds were also identified that presented with a better FaSSIF profile than MU1140, suggesting that the stability profile could be improved with single amino acid substitutions (e.g., Phe1Ile, Arg13Asn and Tyr20Phe). Others have previously identified Arg13 as being prone to proteolytic degradation and have proposed that Arg13Asp may be a substitution capable of inhibiting proteolytic degradation, while conferring a better MIC (Chen et al., 2013). Unexpectedly, the stability profile in serum paralleled, for the most part, the profile in FaSSIF. From this data, it is evident that MU1140 would be expected to remain very stable under the acidic conditions of the stomach, as evidenced by the unmeasurably high levels of stability in FaSSGF. This data also suggests that none of the amino acid substitutions tested affected this inherent property of MU1140. In contrast, the stability in FaSSIF was highly variable amongst compounds, but could clearly be improved with single amino acid substitutions to the point that the resulting compound was unmeasurably stable in this assay. It was concluded from the stability data that the compounds could be ranked according to their half-life: (a) in FaSSIF: Arg13Asn > Tyr20Phe > Phe1Ile > Phe1Leu > MU1140 > Asn18 Ala > Phe17Leu; (b) or in serum: Phe1Ile > Tyr20Phe > Arg13Asn > MU1140 > Phe1Leu > Asn18Ala > Phe17Leu.

Because of the difficulty in identifying *in vitro* assays that may be predictive of *in vivo* efficacy, the top 6 performers in the *in vitro* assays presented above were further tested in animals. We initially focused on *C. difficile* in CDAD as a model of infection because of the relatively better *in vitro* susceptibility profile of the MU1140-variant compounds to this organism. Cannulation was deemed necessary to optimize the time of contact of the compounds with *C. difficile* infected tissues, and to rule out potential complications related to dissolution, transit time, non-specific adsorption, etc. Further, it allowed the side-by-side testing of compounds that were known to be particularly sensitive to trypsin and chemotrypsin degradation (Arg13 of MU1140), which was present in 5 of the 6 compounds tested. This Golden Syrian Hmster model has previously been used to assess the efficacy of *C. difficile* CDAD (Weiss et al., 2014) and is the current standard *in vivo* model used to assess the potential efficacies of agents, including antibiotics, toxin antibodies, and vaccines (Douce and Goulding, 2010; Weiss et al., 2014). In this model, the vehicle control animals demonstrated 100% mortality by day 9, while vancomycin-treated animals are treated with sub-optimal dose targeting 30-60% mortality in the same time frame to ensure the sensitivity of the test system. From this *in vivo* testing, it can be concluded that all six compounds tested showed some signal of efficacy. Nevertheless, a lead compound emerged based on %-survival at day 21: Phe1Ile (100%) > Arg13Asn and Tyr20Phe (66%) > Asn18Ala and Phe17Leu (33%) > Phe1Leu 17%. It is interesting to note that the spore counts and toxin levels (measured post-mortem) paralleled the clinical outcomes. Specifically, OG253-treated animals had no detectable *C. difficile* spores (≤2 Log CFU/g) or Toxin A or B (≤0.27 ng/g) compared to appreciably higher levels observed in vehicle controls (4.09 log CFU, 1061 ng/g Toxin A and 848 ng/g B) and in morbid hamsters. Taken together, this finding may have significant implications on the problem of recurrence with *C. difficile* CDAD.

*Clostridium difficile* and other Gram positive organisms have naturally developed tolerance mechanisms against lantibiotics and other cationic antimicrobial peptides (CAMPs) as an evolutionary response to their ecological niche. These mechanisms include increasing the net positive charge of the cell wall or cell membrane, proteolytic degradation, sequestration, export through efflux pumps, the development of biofilms, immune mimicry, etc. (reviewed in Draper et al., 2015). While tolerance mechanisms have been described for lantibiotic producing strains, only low levels of bonafide resistance have been reported for lantibiotics compared with therapeutic antibiotics; resistance phenotypes have been mostly obtained in the laboratory in order to investigate this phenomenon (Draper et al., 2015). For example, the induction of the *cpr*ACB operon *in vitro* decreases the susceptibility levels of *C. difficile* against several CAMPs (McBride and Sonenshein, 2011; Suárez et al., 2013). However, there is no data available supporting that these genes are actually induced *in vivo* or even relevant to the development of lantibiotic-resistance in humans at levels that would cause these molecules to become ineffective therapeutics. For example, a modest 2–4 fold MIC increase of *C. difficile* to gallidermin to ∼1 μg/ml levels (McBride and Sonenshein, 2011) does not imply that a strain harboring this resistance phenotype would be refractory to gallidermin therapy. While mutants of increased MIC may spontaneously arise at the *cpr* locus (McBride and Sonenshein, 2011), there is no data available on the frequency of such mutation under biologically relevant conditions, nor any data to support that these type of mutations would be selected for in the GI tract of mammalians to a comparable extend as vancomycin-resistant strains of enterococci that are emerging in the clinic (Ahmed and Baptiste, 2017). It is noteworthy to reiterate that nisin resistance was never found to be related to modifications of Lipid II after over 50 years of use as a food preservative.

## Conclusion

This study is the first of its kind to compare a relatively large collection of lantibiotic variants of MU1140 engineered with single amino acid substitutions. These variants were tested for several key properties, in order to define the most “drugable” of those compounds. This triage strategy supported the selection of the top six performers from a library of 418 variants, based on *in vitro* assays, then to a single lead compound (OG253, MU1140-Phe1Ile) based on animal efficacy studies. Of particular clinical relevance, CDI relapse was not observed in hamsters treated with OG253 at the conclusion of the study. While the animal model used is designed to primarily assess efficacy, the data obtained offer some insight on the potential of OG253 to also affect recurrence in patients, based on the absence of relapse that was observed in OG253-treated animals. It was interesting to note that the one *in vitro* assay that paralleled the *in vivo* efficacy data with the greatest accuracy was the stability in serum. It is unclear whether this is an observation with general applicability. The clinical development of OG253 as a *C. difficile* antibiotic will ultimately impinge on the availability of an appropriate formulation that can assure delivery to the distal portion of the ileum and/or colon. Recent advances in enterically coated capsules, micro-encapsulation and other technologies lead us to believe that this is an achievable goal (reviewed in Felton and Porter, 2013; Luo et al., 2016).

## Author Contributions

JK, RS, AD, JX, MP, and WW designed and executed the experiments, and reviewed the manuscript. JP analyzed the data and drafted the manuscript. MH conceptualized, designed the experiments, supervised the study, analyzed the data, and prepared the manuscript.

## Conflict of Interest Statement

JX, MP, and WW had a financial interest in this study as Oragenics sponsored work performed in their respective laboratories. JK and RS, were Intrexon employees during the data collection and initial data analysis. AD was an Oragenics stock-holder and employee during data collection and initial data analysis. JP and MH had a financial interest in Oragenics as stock-holders and employees during the data collection, analysis, and writing of the manuscript. Part of the data contained in this report was presented at the American Society for Microbiology (ASM) Conference on Antibacterial Development held in Washington, DC, United States, on December 11–14, 2016, and is included in pending US applications 62/362,788, 62/362,809, 62/420,328, and PCT/US 17/42206.

## Footnotes

1 Cerep Safety Screen 44: https://www.eurofinsdiscoveryservices.com/catalogmanagement/viewitem/SafetyScreen44-Panel-Cerep/P270

2 http://www.Biorelevant.com

## Supplementary Material

**FIGURE S1 |** Bacterial titers in intestinal tissues. **(A)** Ileum, **(B)** Caecum, and **(C)** Colon.

**FIGURE S2 |** Toxin A and B titers at the outcome of the relapse phase or at euthanasia. Blue bar is Toxin A and Red bar is Toxin B. Limit of Detection (LOD) is 1.6 mg/mL.

**TABLE S1 |** Complete dataset for triage testing.

## Abbreviations

Abu: aminobutyric acid
AviCys: aminovinyl-*D*-cysteine
Dha: 2,3-didehydroalanine
Dhb: 2,3-didehydrobutyrine
Lan: lanthionine
MeLan: methyl-lanthionine
MU1140: Mutacin 1140
