# Supplementary figures and images for: Mutacin 1140 Lantibiotic Variants Are Efficacious Against Clostridium difficile Infection
Source: Front Microbiol. 2018 Mar 16;9:415. doi: 10.3389/fmicb.2018.00415 (PMC5864910; doi:10.3389/fmicb.2018.00415)

A

Ileal CFU/Gram

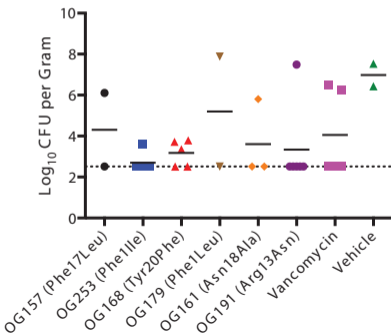

B

Cecal CFU/Gram

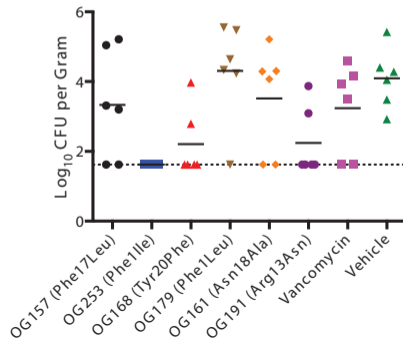

C

Colonic CFU/Gram

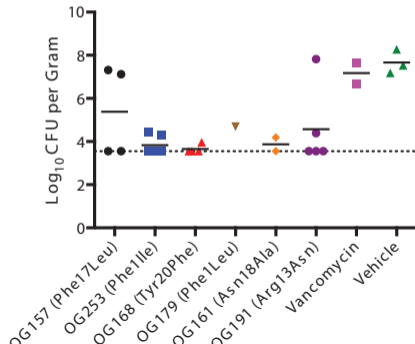

Supplement: Supplementary file 1 [file Data_Sheet_1.PDF]

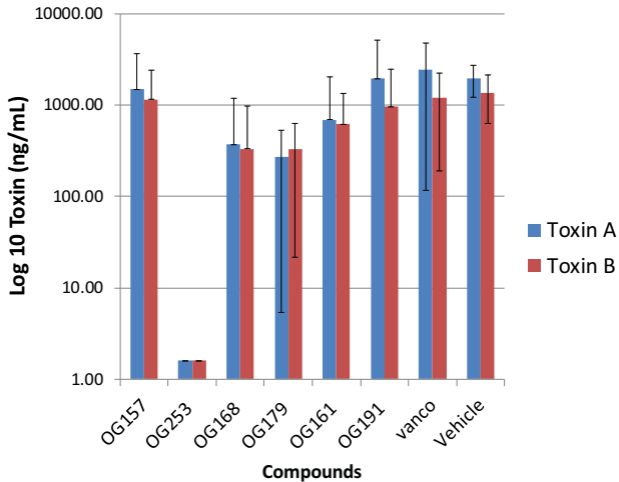

Supplement: Supplementary file 2 [file Data_Sheet_2.PDF]
